# Auxiliary Diagnostic Method for Patellofemoral Pain Syndrome Based on One-Dimensional Convolutional Neural Network

**DOI:** 10.3389/fpubh.2021.615597

**Published:** 2021-04-16

**Authors:** Wuxiang Shi, Yurong Li, Dujian Xu, Chen Lin, Junlin Lan, Yuanbo Zhou, Qian Zhang, Baoping Xiong, Min Du

**Affiliations:** ^1^College of Physics and Information Engineering, Fuzhou University, Fuzhou, China; ^2^Fujian Key Laboratory of Medical Instrumentation & Pharmaceutical Technology, Fuzhou University, Fuzhou, China; ^3^Yida Equity Investment Fund Management Co., Ltd., Nanjing, China; ^4^Department of Mathematics and Physics, Fujian University of Technology, Fuzhou, China; ^5^Fujian Provincial Key Laboratory of Eco-Industrial Green Technology, Wuyi University, Wuyishan, China

**Keywords:** patellofemoral pain syndrome, one-dimensional convolutional neural network, focal loss, attention mechanism, joint angles, surface electromyography

## Abstract

Early accurate diagnosis of patellofemoral pain syndrome (PFPS) is important to prevent the further development of the disease. However, traditional diagnostic methods for PFPS mostly rely on the subjective experience of doctors and subjective feelings of the patient, which do not have an accurate-unified standard, and the clinical accuracy is not high. With the development of artificial intelligence technology, artificial neural networks are increasingly applied in medical treatment to assist doctors in diagnosis, but selecting a suitable neural network model must be considered. In this paper, an intelligent diagnostic method for PFPS was proposed on the basis of a one-dimensional convolutional neural network (1D CNN), which used surface electromyography (sEMG) signals and lower limb joint angles as inputs, and discussed the model from three aspects, namely, accuracy, interpretability, and practicability. This article utilized the running and walking data of 41 subjects at their selected speed, including 26 PFPS patients (16 females and 10 males) and 16 painless controls (8 females and 7 males). In the proposed method, the knee flexion angle, hip flexion angle, ankle dorsiflexion angle, and sEMG signals of the seven muscles around the knee of three different data sets (walking data set, running data set, and walking and running mixed data set) were used as input of the 1D CNN. Focal loss function was introduced to the network to solve the problem of imbalance between positive and negative samples in the data set and make the network focus on learning the difficult-to-predict samples. Meanwhile, the attention mechanism was added to the network to observe the dimension feature that the network pays more attention to, thereby increasing the interpretability of the model. Finally, the depth features extracted by 1D CNN were combined with the traditional gender features to improve the accuracy of the model. After verification, the 1D CNN had the best performance on the running data set (accuracy = 92.4%, sensitivity = 97%, specificity = 84%). Compared with other methods, this method could provide new ideas for the development of models that assisted doctors in diagnosing PFPS without using complex biomechanical modeling and with high objective accuracy.

## Introduction

Patellofemoral pain syndrome (PFPS) is a common knee joint disease in clinical practice, with a prevalence of 10–28% in the general population, about a quarter of the total population, which is often caused by degenerative changes of articular cartilage (1–3). This disease is common in athletes and women, causing severe pain during sports and daily activities, and it affects athletes' careers to a large extent (1, 4). PFPS will have a certain impact on the physical and mental health of patients, making the patients unable to lead an active life (5). Most of the daily activities, that is, up and down stairs, sitting, and squatting, will aggravate the pain of the patients (6). Moreover, PFPS may develop into patellofemoral osteoarthritis (7).

Timely detection and definite diagnosis are the keys to prevent the aggravation of PFPS, but they are not easy (2, 8). Despite the high incidence of PFPS, the pathophysiology of PFPS is unclear (9, 10). Considering that the onset of PFPS is caused by many factors, misjudgment easily occurs (11). At present, the cause of PFPS has two explanations. One is biomechanical joint dislocation, muscle weakness, and excessive joint load around the patella, and the other is pain caused by nerve structure on neurodynamic (6). According to the survey, no clear diagnostic criteria are available at present, but some acceptable reference standards are identified, such as patellar apprehension, patella palpation, patellar apprehension, Waldron test, compression test, and patellar tracking (2). However, these standards are mostly dependent on the subjective judgment of doctors, and the whole diagnosis results and medical effect are strongly related to the rich experience and knowledge of experts, which are not friendly to young doctors. Different standards will lead to different diagnosis results, and no accurate and unified standard is identified for judging PFPS; thus, the diagnostic accuracy is relatively poor (8, 12). Although some PFPS diagnoses in the form of questionnaires (such as the Kujala score) have high sensitivity and specificity, they rely on the subjective answers of the patient and include a certain degree of privacy of the patient, which is difficult for some patients to cooperate (13). At present, invasive or minimally invasive methods are primarily used to assist in the detection of knee injury and diseases. Among the methods, MRI, CT, and other non-invasive detection methods can be more effective in the detection of knee injury and diseases, but these large-scale instruments and equipment are expensive, which are not convenient for daily inspection. As a minimally invasive method, arthroscopy can provide detailed diagnosis information, but repeated incision of the knee joint will cause pain to patients, which is not conducive to the recovery of injury and diseases. Therefore, exploring a new high-precision and low-cost non-invasive PFPS detection method is necessary.

In recent years, increasing studies have focused on the relationship between PFPS and biomechanical parameters (2, 14, 15). Ferrari et al. used the mid-band parameters of surface electromyography (sEMG) to distinguish PFPS by independent *t*-test and other methods (2). Bernard et al. explored whether the coordination of body strength in patients with PFPS has changed (16). Besier et al. used electromyography and lower limb kinematics data to drive a musculoskeletal model and evaluate the muscle strength of PFPS patients and painless subjects during walking and running (17). Myer et al. used a multiple linear logistic regression model to predict the knee-abduction moment when athletes land and explore the relationship between high knee-abduction moment and increased risk of PFPS (18). However, most of the parameters required in these studies are obtained through artificial extraction or the biomechanical model, which is time-consuming. The biomechanical model is based on the musculoskeletal model to establish the relationship between the sEMG signal and joint movement. Nevertheless, the coordination mechanism of the human nerves, muscles, and skeletal system cannot be fully understood, which leads to the inability to accurately simulate the human neuromusculoskeletal system, which causes a fatal flaw in the calculation model, that is, an “individual error.”

Previous studies have shown that when the principle of the system is not clear or unknown, the artificial neural network driven by data has good system characterization and individual adaptability (19). With the development of artificial intelligence technology, artificial neural network methods have been increasingly used in the field of biomechanics and disease diagnosis (20–22). For example, Keijsers et al. used plantar pressure measurements as input to an artificial neural network to classify forefoot pain (23). Otag et al. used an artificial neural network to obtain the ligamentum patellae angle and explained that the prevalence of PFPS in women is greater than that in men based on the difference in angle values between men and women. However, the accuracy in the classification of the left and right knees is mediocre, only 67% (24). Biomechanics will include a variety of non-linear problems, which can be well-solved by an artificial neural network. Thus, this study aims to construct a convolutional neural network (CNN) model to distinguish PFPS through several easy-to-measure biomechanical parameters. Traditional CNN mostly uses two-dimensional convolution, but these biomechanical parameters are generally time series, which have a certain periodicity; thus, this paper proposes to use one-dimensional convolution, causing the filters to only slide on the time axis. Retaining the correlation among various parameters can achieve the time variability of biomechanical parameters and improve the accuracy of network discrimination.

The main contribution of this study is to propose a high-precision, low-cost and easy-to-implement computer-aided diagnostic method, which provides a new idea for the development of a convenient PFPS diagnostic model. The focal loss function is introduced to optimize the network parameters, which improves the balance of the 1D CNN results. By adding attention mechanism into the network and visualizing the output features, we can increase the interpretability of the model to analyze the diversity of biomechanical features involved in PFPS. Moreover, some studies have shown that there are gender differences in PFPS. In this paper, the depth features extracted by one-dimensional CNN are combined with the traditional gender features, and these features are classified through the full connection layer to improve the accuracy of the model.

The rest of this paper is as follows. The second section introduces the data sets and preprocessing methods used in this experiment, and then introduces the neural network model used in this experiment and the experimental environment in detail. In the third section, the experimental results are given and compared. The fourth section discusses the experimental results, and the fifth section summarizes and prospects the full text.

## Methods

### Experimental Data

This study was a retrospective exploratory secondary analysis of a subset of an open data set. This public data set primarily recorded the lower limb kinematic data and sEMG signals of PFPS patients and painless control subjects during walking and running and muscle strength obtained from the musculoskeletal model (17). A total of 27 patients with patellofemoral pain (16 female, 11 male) and 16 painless control groups (eight female, eight male) were included in the study. These patients and painless controls were identified by professional doctors, and they were tested for walking, running, and squatting at a self-selected pace. In this paper, 10 kinds of biomechanical characteristics were selected in walking and running tests, which included three kinds of joint angle values [knee flexion (KF) angle, hip flexion (HF) angle, ankle dorsiflexion (ADF) angle], and seven kinds of sEMG signals [semimembranosus (SEB), rectus femoris (RF), biceps femoris short head (BF), vastus medialis (VM), vastus lateralis (VL), lateral gastrocnemius (LG), and medial gastrocnemius (MG)]. These parameters were selected because they were related to PFPS, which could be measured in real-time without using biomechanical modeling. The original sEMG data used a zero-lag fourth-order recursive Butterworth filter (30 Hz) for high-pass filtering and a Butterworth low-pass filter (6 Hz) for full-wave rectification and filtering. The detailed collection of the entire data set could be found in Reference (17). The experimental data used in this research were obtained from the public data set of this website (https://www.sciencedirect.com/science/article/pii/S0021929009000396?via%3Dihub).

### Data Pre-processing

The data should be cleaned before placing into the neural network. Considering that certain data were missing in the walking and running data of subjects 4 and 43, we eliminated them and tested the data of the remaining 41 subjects, including 26 PFPS patients (16 female, 10 male) and 15 painless controls (eight female, seven male). Each subject had walking and running test data. We combined the data of each subject into a 100 ^*^ 10 matrix to adapt to the input form of a convolutional neural network (100 time-series recorded values, 10 characteristics). The relevant information on subjects is shown in Table 1.

**Table 1:** Mean ± SD age, height, and body mass of subjects.

	**PFPS**		**Controls**	
	**Males (*n* = 10)**	**Females (*n* = 16)**	**Males (*n* = 7)**	**Females (*n* = 8)**
Age (years)	30.5 ± 4.5	28.7 ± 4.6	27.2 ± 3.0	28.8 ± 4.7
Height (m)	1.78 ± 0.08	1.68 ± 0.06	1.80 ± 0.05	1.66 ± 0.05
Mass (kg)	73.5 ± 15.7	62.7 ± 10.0	73.4 ± 18.1	58.3 ± 4.6

The original data had already filtered out the noise, and no filter was needed, but we needed to standardize the parameters of each subject. The range of the joint angle value and EMG signal value was quite different, which was not conducive to the convergence of the neural network; thus, we standardized the range to make it consistent:

(1) $$\begin{array}{l} X_{\text{i}}=\frac{X_{\text{i}}-\bar{X}}{X_{\text{std}}}\;,\; \end{array}$$

where $\bar{X}$ is the mean of each feature of the original data *X*, and *X*_*std*_ is the variance of each feature of the original data *X*.

The preprocessed data were equivalent to a two-dimensional matrix. We flipped the data in the training set horizontally, but we cannot flip such date vertically because the column represented the time axis, which had strong correlation. Therefore, the number of training sets can be doubled, and the performance of the neural network model can be improved.

### Experimental Protocol

**Figure 1:**
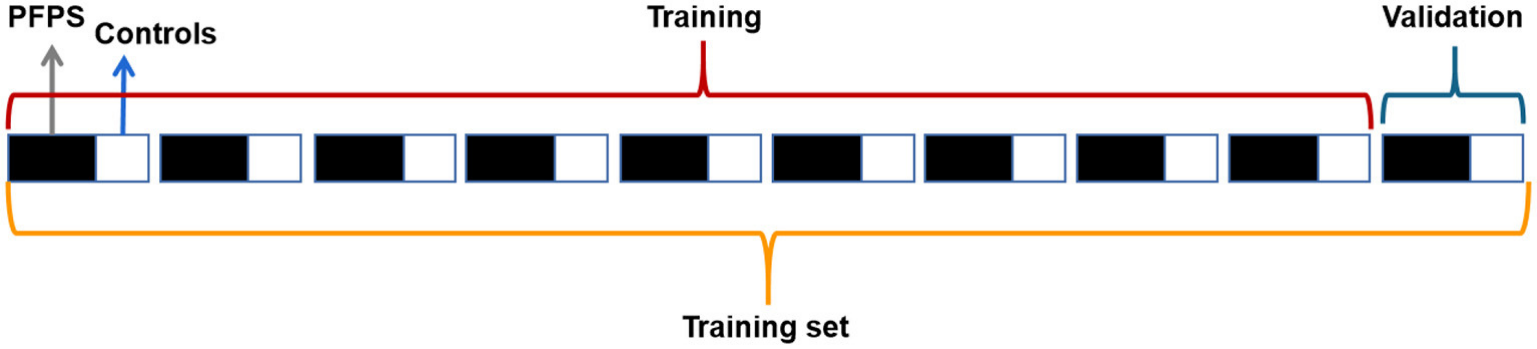
Data partition in 10-fold cross-validation.

We randomly selected 70% of the subjects as the training set and 30% as the test set, and the proportion of PFPS patients and painless controls in the training set was the same as that in the test set. The training set and test set were processed similarly, and then the training set was placed into the neural network for training. Considering that our data set was small and the proportion of PFPS patients was large, we adopted hierarchical 10-fold cross-validation to adjust the network parameters, avoid specificity, and maximize the utilization of data. The training set was equally divided into 10 equal parts, and the proportional relationship between PFPS patients and painless controls in each set was the same. Nine of them were used to train the network, and one was used for verification, which was circularly repeated 10 times to ensure that each copy was used, which is shown in Figure 1.

**Figure 2:**
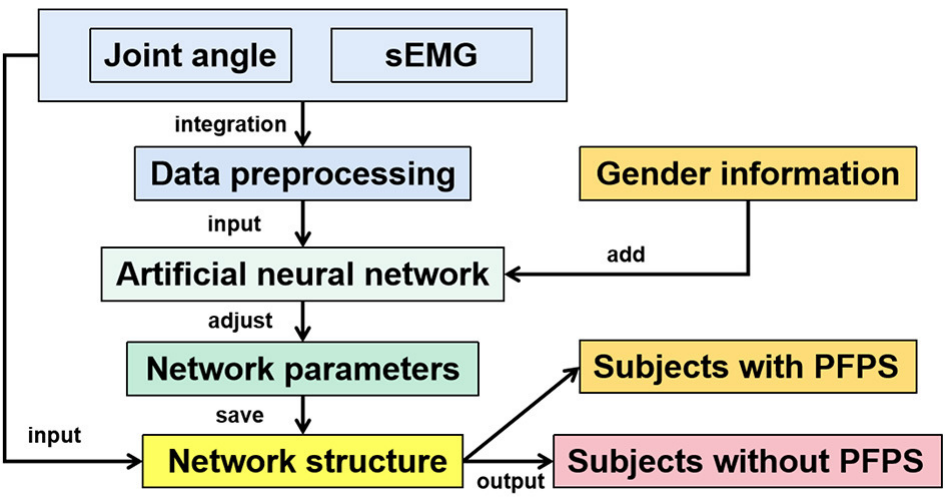
Overall flow chart of the method.

In this paper, several artificial neural network models commonly used in classification tasks were selected for testing, including extreme learning machine (ELM), back propagation neural network (BP), one-dimensional convolution neural network (1D CNN), two-dimensional convolution neural network (2D CNN), long short-term memory (LSTM), VGGNet, and AlexNet. The BP neural network here refers to a fully connected neural network with a hidden layer. This article focused on the 1D CNN, and the other neural networks were primarily used for comparison. Except for VGGNet and AlexNet, all parameters of other artificial neural networks were obtained through 10-fold cross-validation to avoid particularity. The overall flow chart of the method is shown in Figure 2.

### Network Structure

CNN has been proven to have great advantages in a variety of classification tasks, such as image recognition, natural language processing, and human action recognition (25–30). In recent years, a number of excellent CNN classification models have been created, such as AlexNet (31) and VGGNet (32). These two network models belong to the best of their kind, particularly in image classification. In addition, they are often found in medical image classification, which is a good computer-aided diagnostic method. These two models have many parameters. For small data sets, most researchers use transfer learning (33, 34). The data set in this paper is also relatively small, but it is not suitable for transfer learning, because the premise of transfer learning is that the data in the original task and the target task are similar, that is, there is a certain Association for learning. However, most of the training data used in these large-scale classification models such as AlexNet and VGGNet are based on image data, which is very different from the multidimensional time series data in this paper, so it is not applicable.

**Figure 3:**
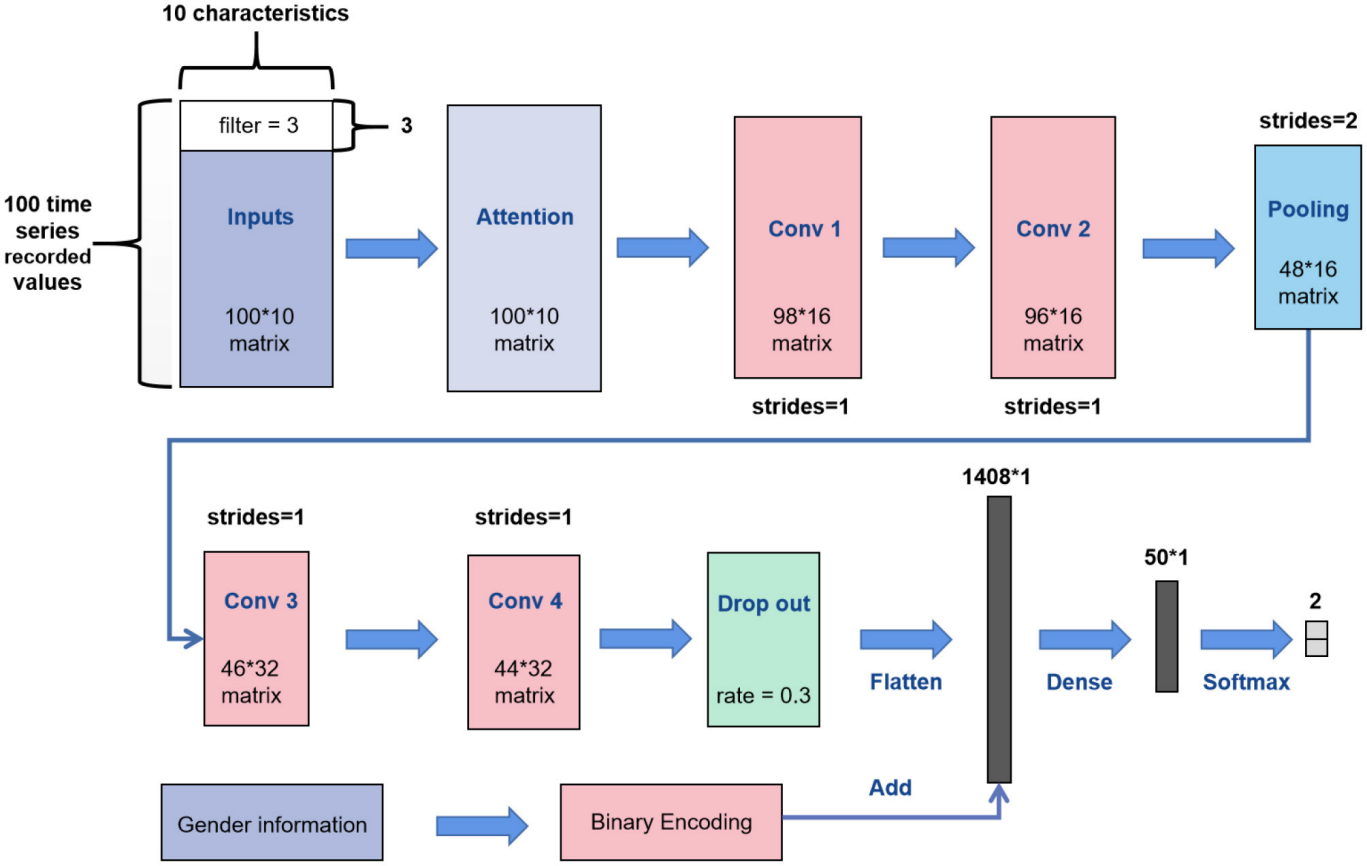
Overall framework of the 1D CNN.

Most of the CNN convolution kernels are two-dimensional. However, according to the characteristics of biomechanical parameters belonging to time-series data, this article utilized the 1D CNN for learning. The network structure of 1D CNN in this paper is shown in Figure 3. We replaced the convolution kernel in the AlexNet model and VGGNet model with one-dimensional convolution kernel to make a better comparison, and other network structures remained unchanged.

Our inputs were the 100 ^*^ 10 matrixes. First, we added a soft attention mechanism to the input, which could reweight the input information adaptively before convolution. This process separated important input features. Then, in the first convolutional layer, we defined 16 filters (also known as feature detector) with the convolution kernel size of 3. The filters only slid on the time axis, and the sliding step size was 1. During training of the first layer, we obtained 16 different feature maps. The structure of the filters in the second convolutional layer was the same as that of the first layer, which was used to learn complex features. The max pooling layer would slide a window of height 2 on the feature map with a step size of 1 and replace it with the maximum value, which discarded half of the value. After the pooling operation, part of the information would be lost; thus, the number of filters in the next two convolutional layers was increased to 32. We added a dropout layer with a dropout ratio of 0.3 (30% of neurons were randomly ignored) after the last convolutional layer to avoid overfitting. Then, we expanded the feature map output of the convolution layer into a one-dimensional vector. Simultaneously, we placed the gender characteristics through binary encoding (01 for males and 10 on behalf of females) and fused such characteristics with the depth feature extracted from the convolution layer. Finally, the fused features were placed into a fully connected neural network with 50 neurons for learning, which were reduced to a vector of length 2 (representing the two types of output) through the softmax activation function. Meanwhile, the optimization algorithm selected Adam and set the learning rate to 0.00001 and the number of iterations to 4,000.

**Figure 4:**
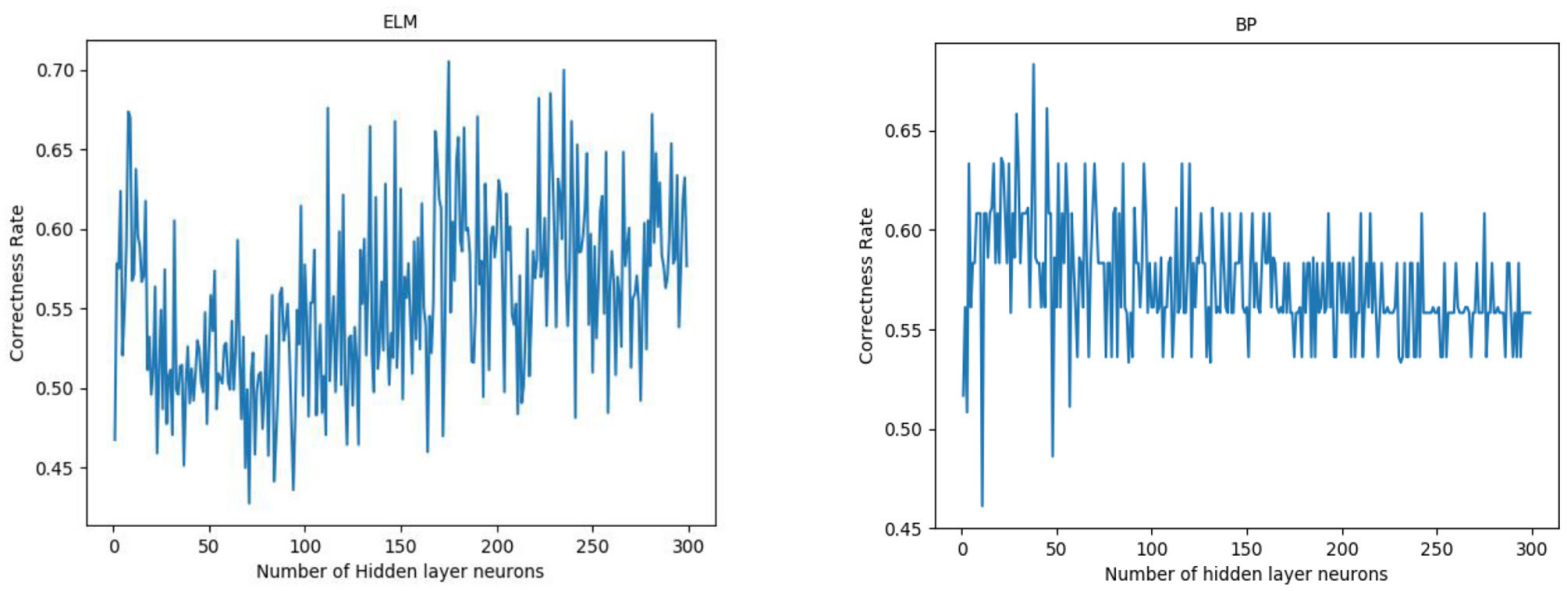
From left to right are the 10-fold cross-validation results of ELM and BP on the running dataset.

The network structure of the 2D CNN was similar to that of the 1D CNN; however, the convolution kernels of the 2D CNN were two-dimensional, which were set to 3 ^*^ 3. This network was designed to facilitate comparison with the 1D CNN. The network structure of ELM and BP only had a single hidden layer. The number of neurons in the hidden layer of ELM and BP was 174 and 37, respectively, which were obtained by ten-fold cross-validation (Figure 4).

**Figure 5:**
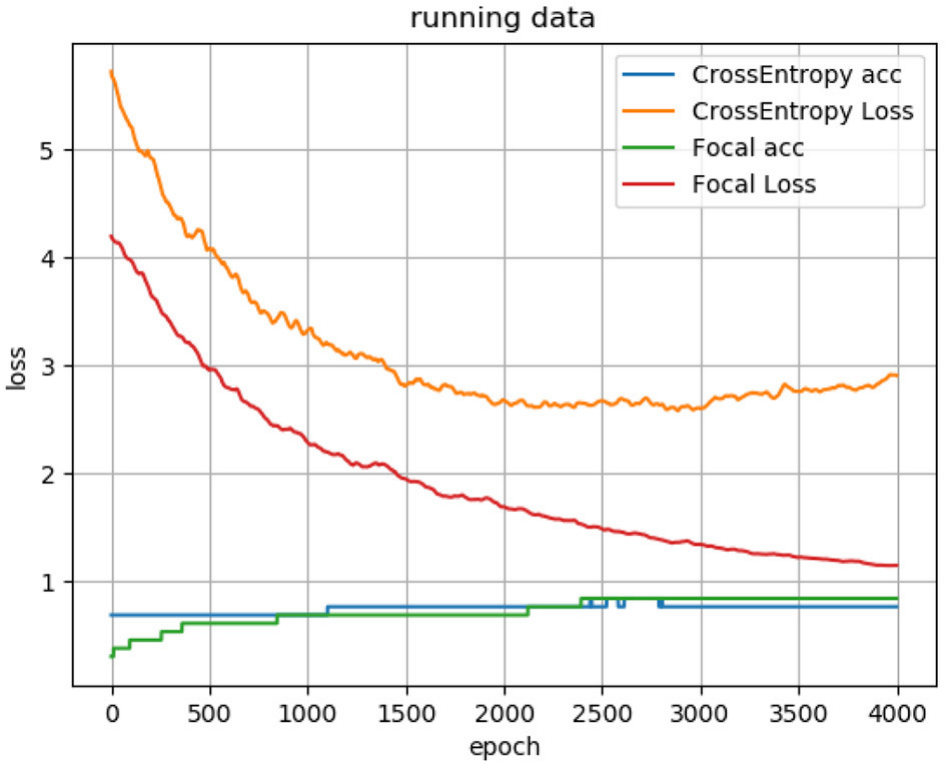
Loss curve and accuracy curve of using focal loss function and cross-entropy loss function for the 1D CNN.

In addition to ELM, other neural networks optimized the parameters by reducing loss. The ordinary cross-entropy loss function was used to optimize the network parameters in most artificial neural networks. Given the large proportion of PFPS tags in the data set, misjudging painless subjects as PFPS by the neural network was easy. Thus, we utilized the focal loss function, which could solve the problem of imbalance between positive and negative samples and reduce the impact of easy-to-predict samples (35, 36):

(2) $$\begin{array}{l} \text{LOSS}=\;-\text{a}{\left(1-{\text{y}}^{\prime }\right)}^{\text{r}*}\text{log}{\text{y}}^{\prime },\;\text{y}=1, \end{array}$$

(3) $$\begin{array}{l} \text{LOSS}=\;-(1-\text{a})\text{y}\prime^{\text{r}*}\text{log}(1-{\text{y}}^{\prime }),\;\text{y}=0, \end{array}$$

where y = 1 is the label of PFPS, and y = 0 is the label of painless control. *y*′is the corresponding predicted label. α is the balance adjustment factor, and *r* is used to control the rate of adjustment. When the sample is easy to predict, that is, *y*′ is larger, its weight 1 − *y*′ will be smaller. Meanwhile, setting *r* > 0 can reduce the loss weight of easy-to-predict samples, which can make the model pay more attention to the difficult-to-predict samples during training. Through many experiments, we set α to 0.2 and r to 2. Moreover, the difference between using the focal loss function and the ordinary cross-entropy loss function for the neural network is shown in Figure 5.

However, ELM does not need to adjust the parameters by iteratively reducing the loss. When the input weight and the bias of the hidden layer are randomly determined, the output matrix of the hidden layer is uniquely determined. The training of the neural network is transformed into solving a linear system:

(4) $$\begin{array}{l} \text{H}\beta =\text{T}, \end{array}$$

where H is the output of the hidden layer node; β is the output weight, and T is the expected output. We can obtain the output weight β by transforming H into the generalized inverse matrix *H*′ and multiplying T.

At present, LSTM is the most popular model in processing time series, which can solve the problem of long-term dependence on information very well. So, this paper also takes this model into account and compares it with 1D CNN. The LSTM model used in this paper consists of 32 basic units.

### Evaluation Indicators

There are many indicators to evaluate the quality of a neural network. However, considering that this research involves the auxiliary diagnosis of diseases, this article used three evaluation indicators, including accuracy (ACC), sensitivity (SES), and specificity (SPC), which were expressed as follows:

(5) $$\begin{array}{l} \text{ACC}=\;\frac{\text{TP}+\text{TN}}{\text{TP}+\text{FP}+\text{FN}+\text{TN}}\;, \end{array}$$

(6) $$\begin{array}{l} \text{SES}=\;\frac{\text{TP}}{\text{TP}+\text{FN}}, \end{array}$$

(7) $$\begin{array}{l} \text{SPC}=\;\frac{\text{TN}}{\text{TN}+\text{FP}}, \end{array}$$

where TP, TN, FN, and FP indicate true positive, true negative, false negative, and false positive, respectively.

In this paper, Keras was used as a deep learning model framework, and TensorFlow was selected as the backend, which created a 1D CNN model. Meanwhile, the experimental environment was CUDA 10.1; the GPU was NVIDIA GeForce GTX 1080; the CPU was Intel Core i7-8700, and the operating system was Windows 10.

## Results

We tested each model on three different data sets of the subjects, including walking data, running data, and the combination of walking and running data to explore the pros and cons of the models as a whole. The three data sets were divided similarly, and 70% of the data sets were randomly selected for training, and the training data were subjected to 10-fold cross-validation to obtain the optimal model parameters. Then, the remaining 30% of the data were used for testing. Considering that our data set was small, the batch size of the network was set to the entire training set. Using this method, the loss direction determined by the full data set could represent the sample population, thereby moving accurately toward the direction of the extreme value.

We repeated each experiment 10 times independently and took the average of the results as the judgement of the model. For the data division of each trial, the data distribution in the training set and test set was the same.

### Comparison Results of all Neural Network Models

**Figure 6:**
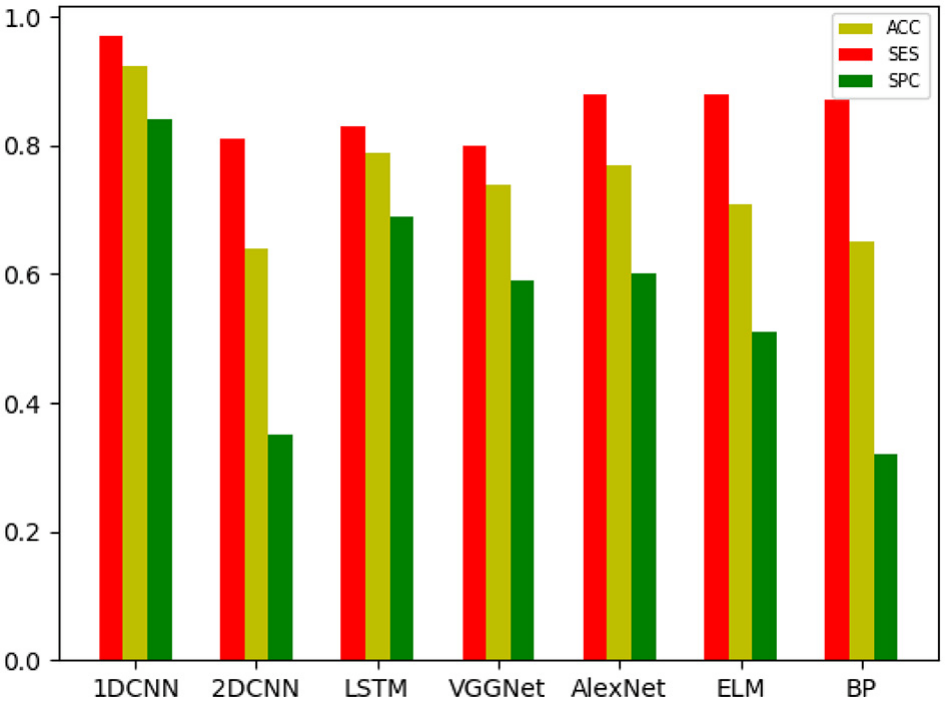
The results of each neural network on the running data set.

The overall results are shown in Tables 2–4. It can be seen from the figure that all the neural network models have the best effect on the running data set. In order to make the comparison results on the running data set more visible, this paper makes a histogram, as shown in Figure 6.

**Table 2:** Results on walking data set.

**Network models**	**ACC**	**SES**	**SPC**	**Training time(s)**
1D CNN	0.68	0.77	0.53	43.8
2D CNN	0.61	0.81	0.29	158
LSTM	0.63	0.73	0.51	153.1
VGGNet	0.61	0.91	0.14	1913
AlexNet	0.61	1.00	0.00	800.7
ELM	0.66	0.89	0.29	0.02
BP	0.58	0.59	0.55	4.32

**Table 3:** Results on running data set.

**Network models**	**ACC**	**SES**	**SPC**	**Training time(s)**
1D CNN	0.924	0.97	0.84	43.7
2D CNN	0.64	0.81	0.35	157.4
LSTM	0.79	0.83	0.69	152
VGGNet	0.74	0.80	0.59	1912.4
AlexNet	0.769	0.88	0.60	800.5
ELM	0.71	0.88	0.51	0.03
BP	0.65	0.87	0.32	4.42

**Table 4:** Results on combined walking and running data set.

**Network models**	**ACC**	**SES**	**SPC**	**Training time(s)**
1D CNN	0.77	0.77	0.70	43.8
2D CNN	0.615	0.88	0.20	160
LSTM	0.76	0.90	0.58	155
VGGNet	0.62	0.80	0.42	1914
AlexNet	0.76	0.84	0.64	801
ELM	0.59	0.82	0.20	0.03
BP	0.56	0.66	0.40	4.51

### Results of Attention Mechanism

**Figure 7:**
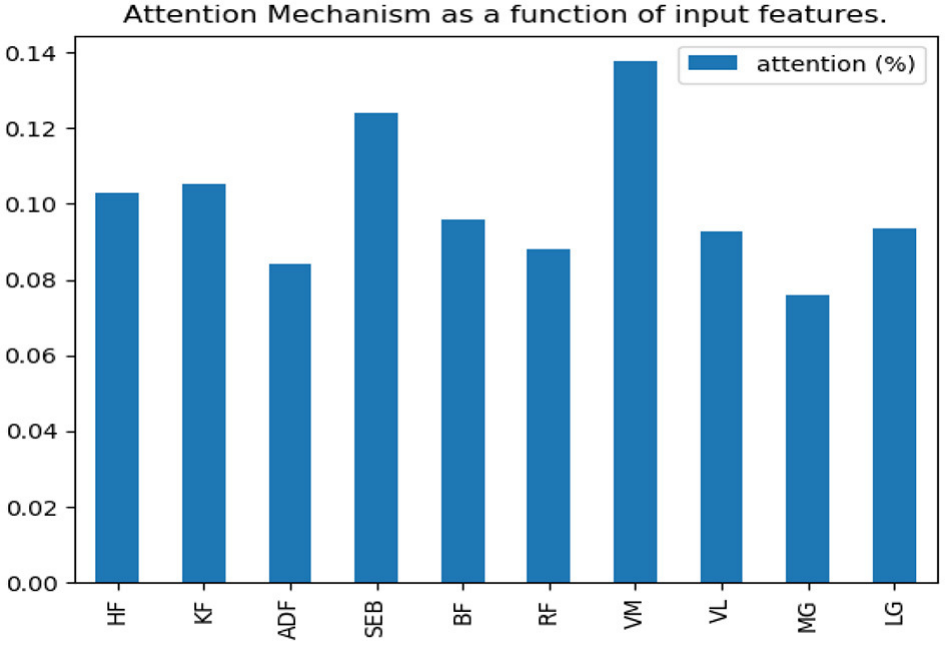
Attention probability distribution of input features on running data set.

According to the comparison results in the previous section, this paper will make further research on the running data set. The soft attention mechanism could reweight all information adaptively before aggregation. Consequently, important information could be separated, and the interference of unimportant information could be avoided to improve the accuracy. In this study, the weight of time dimension was fixed, and only the input feature dimension was weighted. After the neural network model was trained, the weight of feature dimension was determined. Finally, we visualized the weight assigned to each feature by the attention mechanism and observed the features that belonged to the key features (Figure 7).

### Visualization Results of the CNN Model

**Figure 8:**
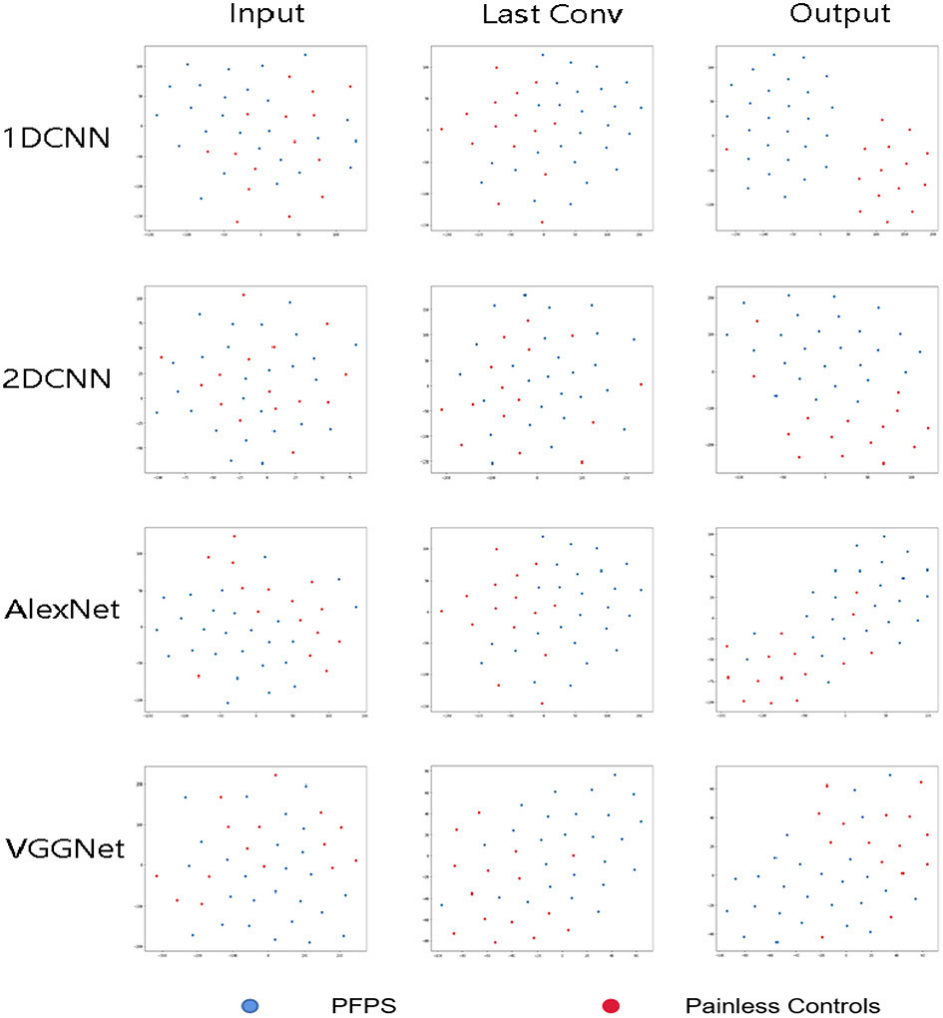
Visualization of feature representations extracted from input layer, last convolutional layer and output layer for running data set.

In this section, the T-SNE method was used to visualize the feature distribution of the input layer, final convolution layer, and output layer of the four CNN models for running data set. In this way, we can easily compare the ability of learning features from the original biomechanical data among different CNN models Figure 8).

## Discussion

As shown in Tables 2–4, all the neural network models perform best in the running data set, which indicates that PFPS will have a significant impact on the lower limb biomechanical features of patients during running. Pain is a protective mechanism for patients, and patients will take corresponding compensatory behavior to complete the exercise to reduce pain, thereby resulting in changes in biomechanical features. The task intensity of running is higher than that of walking, which may lead to evident compensatory changes in patients with pain, thereby making the neural network easier to learn.

By adding attention mechanism into the 1D CNN model and outputting the weight results of attention mechanism, we ranked the importance of biomechanical features in identifying PFPS and determined the biomechanical features that were important for the identification of PFPS. As shown in Figure 6, the three most concerned features of the neural network are VM, SEB, and KF. However, whether the changes of these biomechanical features cause PFPS, or whether the pain of PFPS causes the changes of these biomechanical features, that is, whether these biomechanical features are risk factors for PFPS, remain unclear.

All neural network models have high specificity and low sensitivity. There are two reasons for this result. First, more PFPS patients are included in the data set, which makes the learning of the network prone to deviation. Second, the data set is relatively small, which makes the neural network easy to overfit. Previous studies have shown that CNN tends to perform better in big data. In the case of a larger data set, we hypothesize that the accuracy of our model can be improved. In addition, although ELM and BP are feedforward neural networks with a hidden layer, the number of hidden layer nodes is different when they reach the optimal situation probably because their network weights are determined in different ways. ELM directly determines the weight of neurons in the hidden layer by solving the generalized inverse matrix, whereas BP gradually determines the weight of neurons in the hidden layer by back propagation.

In all data sets, the 1D CNN performs best, particularly on the running data set (ACC = 0.924, SES = 0.97, SPC = 0.84). Meanwhile, the comparison of the classification results shows that the 1D CNN is suitable for the characteristics of these biomechanical parameters than the 2D CNN. In addition, the introduction of focal loss does not greatly improve the accuracy of the neural network, but it makes the neural network easier to learn to ensure that the SES and SPC values will not differ remarkably. The results of 1D CNN are also better than LSTM, which may be because 1D CNN pays more attention to the feature changes in local time period, while LSTM is more suitable for data with long-term dependence. The disease detection of pain type should pay more attention to the instant changes caused by pain. Moreover, because LSTM adopts full connection computing mode, its computation is very time-consuming, resulting in poor real-time performance. Compared with the LSTM model, the local connection and weight sharing mechanism of the 1D CNN model reduces a large number of network parameters, so that the model can train faster and reduce the risk of overfitting.

In this paper, the *t*-SNE method was used to reduce the dimension and visualize the features extracted from the CNN model and determine whether the features extracted from the neural network model were separable, which increased the interpretability of the model. As shown in Figure 7, the 1D CNN model constructed in this paper could easily obtain segment able features.

## Conclusion

This paper proposed a method to assist the diagnosis of PFPS through the 1D CNN model. Different from previous studies, this method does not require complex biomechanical models, and it can achieve high accuracy (ACC = 0.924) only through some directly measurable biomechanical parameters and the gender of subjects. This method is easy to operate. After the neural network has learned a certain number of features, the model is saved. Then, the PFPS can be intelligently determined by the neural network in real-time through the lower limb joint angle values and sEMG signals of subjects in a gait cycle. This prospective study provides new insights into the auxiliary diagnosis of PFPS, which can be used to develop a convenient, efficient, and universal auxiliary diagnosis model for PFPS.

Compared with previous research (2, 37), the method of this study has higher sensitivity (SES = 97%), and specificity (SPC = 84%). Ferrari et al. used the mid-band parameters of sEMG, which were associated with anterior knee pain to determine PFPS. The method had 70% sensitivity and 87% specificity, and the trial involved 51 subjects, including 22 PFPS patients and 29 painless controls (2). Briani et al. used the sEMG signal of VM to diagnose PFPS, and obtained 72% sensitivity and 69% specificity, and obtained 68% sensitivity and 62% specificity through the sEMG signal of VL. The trial involved 59 subjects, including 31 patients with PFPS and 28 painless controls (37).

This study is a preliminary investigation, and its applicability requires caution. This study has some limitations, which need to be considered in future studies. For example, a comparative experiment should be conducted to explore whether these biomechanical changes caused by pain or PFPS caused by these biomechanical changes. Another limitation is that the data set of the paper is relatively small, and the convolutional neural network often performs better on large data sets; therefore, larger sample size must also be considered in the next work. Meanwhile, future work must focus on the specific subclassifications of PFP diagnoses.

## Data Availability Statement

The original contributions presented in the study are included in the article/Supplementary Material, further inquiries can be directed to the corresponding author/s.

## Author Contributions

WS, BX, and MD conceived the layout, rationale, and plan of this manuscript. WS wrote the first draft of the manuscript. CL, JL, YZ, QZ, DX, and YL edited the manuscript. All authors have read and agreed to the published version of the manuscript.

## Conflict of Interest

The authors declare that the research was conducted in the absence of any commercial or financial relationships that could be construed as a potential conflict of interest.
